# Avian influenza viruses at the wild–domestic bird interface in Egypt

**DOI:** 10.1080/20008686.2019.1575687

**Published:** 2019-02-20

**Authors:** Mahmoud M. Naguib, Josanne H. Verhagen, Ahmed Samy, Per Eriksson, Mark Fife, Åke Lundkvist, Patrik Ellström, Josef D. Järhult

**Affiliations:** a Zoonosis Science Center, Department of Medical Biochemistry and Microbiology, Uppsala University, Uppsala, Sweden; b Zoonosis Science Center, Department of Medical Sciences, Uppsala University, Uppsala, Sweden; c National Laboratory for Veterinary Quality Control on Poultry Production, Animal Health Research Institute, Giza, Egypt; d Centre for Ecology and Evolution in Microbial Model Systems, Linnaeus University, Kalmar, Sweden; e Genetics and Genomics, The Pirbright Institute, Surrey, UK

**Keywords:** LPAIV, HPAIV, AIV, IAV, migration, wild birds, epidemiology, ecology, H5N1, Africa

## Abstract

Wild birds of the orders Anseriforme*s* (mainly ducks, geese and swans) and Charadriiformes (mainly gulls, terns and waders) constitute the natural reservoir for low pathogenic avian influenza (LPAI) viruses. In Egypt, highly pathogenic avian influenza (HPAI) H5N1 and LPAI H9N2 viruses are endemic in domestic poultry, forming a threat to animal and human health and raising questions about the routes of introduction and mechanisms of persistence. Recently, HPAI H5N8 virus was also introduced into Egyptian domestic birds. Here we review the literature on the role of wild birds in the introduction and endemicity of avian influenza viruses in Egypt. Dabbling ducks in Egypt harbor an extensive LPAI virus diversity and may constitute the route of introduction for HPAI H5N1 and HPAI H5N8 viruses into Egypt through migration, however their role in the endemicity of HPAI H5N1, LPAI H9N2 and potentially other avian influenza virus (AIV) strains – by means of reassortment of viral genes – is less clear. Strengthened surveillance programs, in both domestic and wild birds, that include all LPAI virus subtypes and full genome sequencing are needed to better assess the wild–domestic bird interface and form a basis for evidence-based measures to limit and prevent AIV transmission between wild and domestic birds.

## Introduction

In 2006, highly pathogenic avian influenza (HPAI) viruses of the H5N1 subtype originating from China were detected in birds in Egypt. Upon introduction, HPAI H5N1 viruses became endemic in the population of domestic birds in Egypt [1]. Similarly, in 2010 the Asian G1-like lineage of low-pathogenic avian influenza (LPAI) H9N2 viruses were detected in birds in Egypt and became endemic in the population of domestic birds [2]. Furthermore, in 2016 HPAI viruses of the H5N8 subtype originally from China (but likely having gone through several steps of transmission in Asia and Europe) were detected in birds in Egypt [3,4]. The presence of HPAI H5N1 and LPAI H9N2 viruses in domestic birds in Egypt represents a threat to animal and human health and raises questions about the routes of introduction and the mechanisms of persistence. The co-circulation of three avian influenza virus (AIV) subtypes in domestic birds and the zoonotic potential of HPAI H5N1 and LPAI H9N2 viruses highlights Egypt as a hotspot for generation of new sub- and genotypes of AIVs [5].

Wild birds of the orders Anseriformes (mainly ducks, geese and swans) and Charadriiformes (mainly gulls, terns and waders) are the natural reservoir for LPAI viruses. Based on the virus surface glycoproteins hemagglutinin (HA) and neuraminidase (NA), the viruses as found in birds are classified into 16 HA subtypes and 9 NA subtypes [6], that occur in numerous combinations such as H5N1 and H9N2. The viral genome consists of eight gene segments encoding 12 different proteins, including HA and NA. The segmented virus genome enables evolution by mixing of gene segments from two or more influenza A viruses, so called genetic reassortment. In general, LPAI viruses do not cause disease in wild or domestic birds. However, viruses of the H5 and H7 subtype can evolve into HPAI viruses upon introduction into poultry, causing up to 100% mortality in poultry [7,8]. HPAI outbreaks in poultry have been controlled rapidly by methods such as mass culling. Yet, HPAI H5N1 viruses that share a common ancestral virus (A/Goose/Guangdong/1/96, GsGd), have continued to circulate and cause outbreaks in poultry in several regions of the world including Egypt. These outbreaks were associated with human infections and spillback to wild birds.

What is the role of wild birds in the epidemiology and evolution of HPAI H5N1 and LPAI H9N2 in domestic birds in Egypt? The ability of wild birds – in particular waterfowl – to replicate a high diversity of AIV and to be highly mobile, facilitates the emergence of newly introduced and/or newly formed AIV. The long-distance spread of HPAI H5N1 viruses has been associated with migratory bird movements [9], including the introduction of HPAI H5N8 viruses into North America in 2016 with waterfowl [10]. In addition, North American wild birds amplified AIV during summer breeding seeded by overwintering virus persisting locally and virus introduced from a wide range of latitudes [11]. Once introduced and adapted to poultry, viruses are able to keep circulating among domestic bird populations. Yet, wild birds can affect AIV epidemiology and evolution in domestic birds locally through the influx of new AIV genes via reassortment and introduction of new subtypes. Here we investigate, based on available literature, the role of wild birds in AIV introduction and persistence in Egypt.

## AIV in domestic birds in Egypt

In Egypt, a high density of domestic birds are housed in different production types including large commercial farms (industrial and integrated farms), non-regulated non-registered small- to medium-scale farms, and backyard farms (i.e. house-holdings). Non-regulated non-registered small- to medium-scale farms produce more than 75% of the broilers in Egypt [12]. Host species distribution among registered commercial farms favours chicken (>80%), followed by ducks, geese, and turkeys [13]. In addition, quails and pigeons are kept as domestic birds. In most studies, domestic birds were sampled at farms during outbreaks or at live bird markets (LBM). Domestic birds were sampled for virus detection using cloacal, oropharyngeal and organ swabs [2,14,15]. In Egypt, little is known about the occurrence of other AIV subtypes than H5, H7 and H9 in asymptomatic or sick domestic birds [2,14–16]. Globally, LPAI virus surveillance activities in domestic birds have been mostly limited to the detection of H5, H7 and/or H9 viruses, however a high subtype diversity can be found in domestic birds when investigated [17–20]. For instance, LPAI viruses of the subtypes H3N2, H3N6, H3N8, H4N6, H6N2, H6N6, H7N3 and H9N2 have been isolated from domestic ducks sampled in Vietnam [18]. Also, LPAI viruses of the subtypes H3N2, H3N8, H4N2, H4N6, H6N2 and H9N2 have been isolated from ducks at LBM in Korea [20], and H1N2, H1N3, H3N6, H4N2 and H10N7 have been isolated from ducks at markets in Bangladesh [19]. LPAI viruses of the subtypes H1N1, H1N5, H6N1, H6N5, H7N1, H7N4, H7N7, H8N4, H9N2 and H10N7 have been isolated from turkeys or chickens during outbreaks at farms in the Netherlands [17]. Thus, AIV sub- and genotype diversity in domestic birds in Egypt may well be higher than currently described.

HPAI H5N1 viruses were first detected in domestic birds in Egypt in 2006 [14]. Despite efforts to eliminate HPAI H5N1 virus from poultry in Egypt, the virus still remains deeply entrenched throughout the country infecting many avian species, including chicken, duck, turkey, goose, and quail in both commercial and backyard farms [1], and is since 2008 considered endemic in domestic birds. Genetic changes of the HA protein resulted in the co-circulation of genetic clades 2.2.1 and 2.2.1.1 from late 2009 to 2011. The 2.2.1.1 clade is thought to have emerged as a vaccine-escape mutant [1,21]. Clade 2.2.1 of the HPAI H5N1 viruses continued to evolve to form a new phylogenetic cluster named clade 2.2.1.2, which recently evolved into clade 2.2.1.2a [22]. This has been associated with an increased number of human infections with HPAI H5N1 virus [22]. Internal gene segments of these H5 viruses were genetically closely related to isolates from Asia. Also, a newly introduced HPAI virus of the H5N8 subtype, genetically related to HPAI H5N1 GsGd virus and belonging to clade 2.3.4.4 has been reported in Egypt in early 2017 in domestic ducks in backyard farms and at commercial chicken farms [23]. HPAI H5N8 virus internal segments were genetically closely related to AIV from Europe and Asia [24].

In addition to the endemic status of HPAI H5N1 virus in poultry, Egypt has experienced incursions of LPAI H9N2 viruses in 2010 [2,25]. LPAI H9N2 viruses were detected in chickens, ducks, turkeys, quail, and pigeons in both commercial and backyard farms in northern Egypt [2]. Based on HA sequence analysis, these viruses were phylogenetically related to isolates from Lebanon, Bangladesh, and Israel [26]. Reassortment of LPAI H9N2 virus with AIV gene segments from wild birds that originate from in or outside Egypt was observed based on full genome analyses of H9N2 virus isolates from pigeons [27]. Despite LPAI H9N2 being endemic in Egypt, the highest H9N2 incidence in domestic birds in the Nile Delta was observed in January 2012, 2013 and 2014 [25,28]. In Egypt, there is evidence for year-round AIV detection in backyard poultry, while AIV infections in backyard and commercial farms seem to peak in winter and spring [15,29].

## Migratory flyways of waterfowl crossing Egypt

With its position in northeastern Africa, Egypt constitutes part of a land bridge between Africa and Eurasia [30]. This is reflected in migratory flyways of birds, where Egypt connects the Palearctic with the Afro-Tropical realms. In Egypt, the Mediterranean-Black Sea and East Africa-West Asia flyways overlap with the more regional Rift Valley-Red Sea flyway [31]. Within the Rift Valley-Red Sea flyway, migratory birds from Europe and Asia follow the eastern Mediterranean coast or the Jordan Valley to northern Egypt and then winter along the Mediterranean coast, Nile Valley or Red Sea coast, or continue southwards towards the East African Rift Valley and Sub-Saharan Africa. This flyway is used by a wide variety of bird species including bird species of the order Anseriformes and Charadriiformes [31].

**Figure 1.**
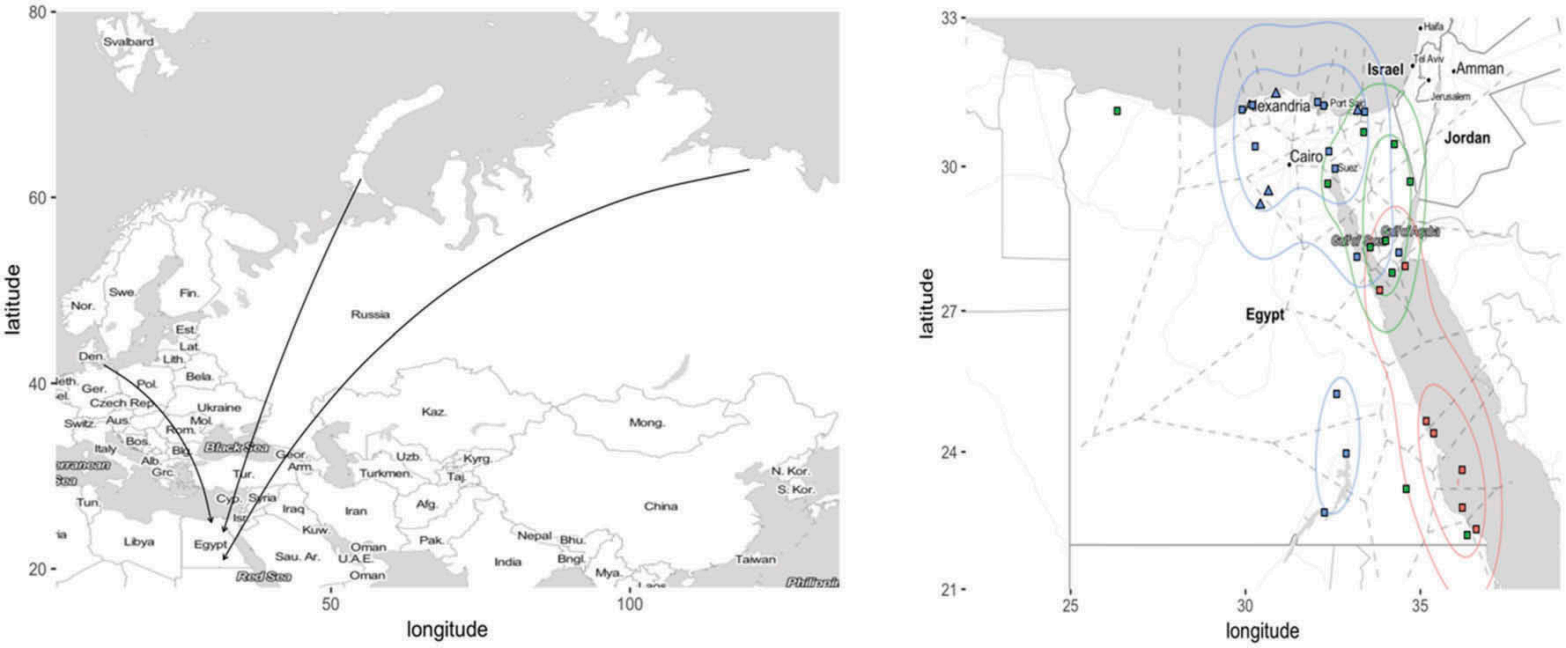
Migratory flyways and important bird sites in Egypt.Left pane: Schematic drawing of avian migratory flyways connecting the Palearctic and the Afro-Tropical realms, including the Rift Valley/Red Sea flyway. The arrows are rough indicators of how birds originating from various parts of Eurasia concentrate towards Egypt during migration. Right pane: Important wild bird areas in Egypt. Ramsar sites are indicated by triangles and Important Bird and Biodiversity Areas (IBAs) are indicated by rectangles. Wetland habitats are indicated with blue, scrub habitats are indicated with green and offshore islands are indicated with red. Dashed lines divide Egypt into regions around each important wild bird site (based on Voronoi tessellation). Colored contour lines indicate density distribution of important bird sites in Egypt based on three habitat types: ‘scrub/wadi/desert’, ‘offshore island’ and ‘wetland’.

The most important areas for waterfowl in Egypt are the wetlands located along the Mediterranean coast and along the Nile delta and valley [31–34]. Along the Nile Valley and the Red Sea coast 34 important bird and biodiversity areas (IBAs) have been identified by BirdLife International [34], including four internationally recognized stopover sites for migratory birds (so called Ramsar sites [33]). The IBAs are categorized into scrub/wadi/desert, offshore islands, and wetlands. On the offshore islands; gulls, terns and herons breed in large colonies. Gulls and terns may conduct longer foraging trips inland during the breeding season that may affect AIV epidemiology locally [35–39]. The wetlands are most important for AIV epidemiology due to aggregation of waterfowl, gulls and shorebirds [40] (Figure 1).

Birds of the order Anseriformes and Charadriiformes – in particular dabbling ducks, gulls and shorebirds – are the most frequent AIV carriers in the northern hemisphere, with peak prevalence during the fall [41–43]. Particularly, several of the *Anas* ducks and their allies (i.e. dabbling ducks), with breeding sites in central-northern Eurasia, winter along the Mediterranean coast or in the Nile Delta or upper Nile Valley. This is especially true for northern shoveler (*Spatula clypeata*), Eurasian green-winged teal (*Anas crecca crecca*) and Eurasian wigeon (*Mareca penelope*) [32,44,45]. These birds arrive in Egypt from September and onwards and may carry AIV upon arrival, which can be further transmitted to resident wild waterfowl that share mutual habitats with the migratory dabbling ducks. In addition, the wetlands in the Nile Delta region are rich in mudflats attracting e.g. long-distance high arctic shorebirds including species such as ruddy turnstone (*Arenaria interpres*) [32]. Ruddy turnstones have earlier been suggested to contribute to the local amplification of AIVs in North America [46]. Taken together, several avian species being well-described AIV hosts with breeding areas from various parts of Eurasia, overwinter in Egypt. Thus, there is a considerable risk that these avian species introduce AIVs into Egypt on their arrival during fall migration and that these AIV – partially or completely – transmit to resident related avian species with further transmission to local domestic birds.

## AIV in wild birds in Egypt

A high diversity of AIV subtypes have been detected in wild birds sampled in Egypt. In the 1970s, AIVs of subtypes H3N1, H4N1, and H11N6 were reported in Egypt among migratory waterfowl [47,48]. In response to the emergence of HPAI H5N1 virus in China, wild birds in Egypt have been sampled for AIV, in particular for HPAI H5N1 virus, from 2003 onwards. In most studies, wild birds were sampled after capture using mist nets or after being shot, or later at LBM. Some studies reported the sampling of diseased or dead wild birds from LBM [3]. Wild birds were sampled for virus detection using cloacal swabs, and in some cases additional oropharyngeal swabs. As a result, most known HA subtypes have been isolated from wild birds (Table 1). In total, 23 subtype combinations have been detected as shown in Table 1. AIVs of subtypes H7 and H10 were most frequently isolated from wild birds [49,50]. They have been reported in combination with different NA subtypes: H7N1, N3, N7, and N9, and H10N1, N4, N7, and N9. Other HA subtypes isolated from wild birds in Egypt were H1, H3, H4, H5, H6, H9, H11 and H13 (Table 1). Despite the isolation of these subtypes, the majority of wild bird surveillance studies in Egypt focus on H5 detection, and in some cases H7 and H9 detection. Furthermore, a high number of samples from birds that tested AIV-positive, but H5-negative were not further investigated. For example, 201/1304 birds in [51]; 732/7894 birds in [50]; 703/7678 birds in [49], and moreover, birds were tested exclusively for H5 [52]. Nevertheless, the AIV-positive H5-negative birds may affect AIV epidemiology and evolution in domestic birds in Egypt directly by spillover/spillback of AIV, or indirectly by genetic reassortment of AIV circulating in domestic birds.

**Table 1.** Avian influenza viruses isolated from wild birds sampled in Egypt during 2003–2018. The virus pathotype (highly pathogenic avian influenza [HPAI], low pathogenic avian influenza [LPAI] or not determined [n/d]) are indicated for viruses of the H5 subtype, remaining viruses were LPAI.

Subtypes	Species	Year	Reference
H1N1	Northern shoveler (*Spatula clypeata*)	2003; 2005; 2006	[50]
	Eurasian green-winged teal (*Anas crecca crecca*)	2004; 2007	[50]
	Northern pintail (*Anas acuta*)	2012	[17]
H1N2	Eurasian green-winged teal (*Anas crecca crecca*)	2003	[50]
H2N8	Northern shoveler (*Spatula clypeata*)	2007	[50]
H3N1	Wild bird	1970s	[47]
H3N8	Ducks	1990s	[57]
H4N1	Ducks	1970s	[47]
H4N6	Ducks	1976	[48]
	Eurasian green-winged teal (*Anas crecca crecca*)	2005	[50]
H5Nx	Rock dove (*Columbia livia*)	2005 n/d	[58]
H5N1	Great egret (*Ardea alba*)	2006 HPAI	[50]
	Eurasian green-winged teal (*Anas crecca crecca*)	2005 LPAI & HPAI	[51]
	Cattle egret (*Bubulcus ibis*)	2014 HPAI	[60]*
	Crow (*Corvus sp*.)	2007 HPAI	[60]*
H5N2	Northern shoveler (*Spatula clypeata*)	2003 LPAI	[50]
H5N8	Eurasian coot (*Fulica atra*)	2016 HPAI	[3]
	Eurasian green-winged teal (*Anas crecca crecca*)	2016 HPAI	[55] [60*]
H6N2	Northern shoveler (*Spatula clypeata*)	2003; 2006	[15,50]
	Whiskered tern (*Chlidonias hybrida*)	2004	[60*]
	Wild duck	2005	[66]
	Eurasian green-winged teal (*Anas crecca crecca*)	2006	[50]
H7Nx	Mallard (*Anas platyrhynchos*)	2012	[60*]
	Northern shoveler (*Spatula clypeata*)	2010	[60*]
H7N1	Northern shoveler (*Spatula clypeata*)	2004; 2012	[17,50]
H7N3	Northern shoveler (*Spatula clypeata*)	2006; 2007	[50]
H7N7	Eurasian green-winged teal (*Anas crecca crecca*)	2004	[50]
	Northern shoveler (*Spatula clypeata*)	2004	[50]
	Black Kite (*Milvus migrans*)	2005	[59]
	Egyptian Goose (*Alopochen aegyptiaca*)	2006	[50]
H7N9	Northern shoveler (*Spatula clypeata*)	2006; 2007	[50]
H9N9	Northern pintail (*Anas acuta*)	2005	[50]
H10N1	Eurasian green-winged teal (*Anas crecca crecca*)	2003; 2005	[50]
	Northern shoveler (*Spatula clypeata*)	2006	[50]
H10N4	Northern shoveler (*Spatula clypeata*)	2007	[50]
H10N7	Northern shoveler (*Spatula clypeata*)	2004; 2007	[50],[60*]
	Eurasian green-winged teal (*Anas crecca crecca*)	2005; 2007	[50]
	Mallard (*Anas platyrhynchos*)	2012	[17]
	Northern pintail (*Anas acuta*)	2012	[17]
H10N9	Northern shoveler (*Spatula clypeata*)	2006	[50]
	Eurasian green-winged teal (*Anas crecca crecca*)	2007	[50]
H11N6	Wild duck	1976	[48]
H11N9	Eurasian green-winged teal (*Anas crecca crecca*)	2004	[50]
H13N8	Eurasian green-winged teal (*Anas crecca crecca*)	2005	[49]

*Virus isolate not described but data was obtained from the NIAID Influenza Research Database (IRD) [60] through the website at http://www.fludb.org

In Egypt, most AIV subtypes were found in northern shoveler with 12 different AIV subtypes (i.e. H1N1, H2N8, H5N2, H6N2, H7N1, H7N3, H7N7, H7N9, H10N1, H10N4, H10N7, and H10N9) and in Eurasian green-winged teal with 11 different AIV subtypes (i.e. H1N1, H1N2, H4N6, H5N1, H6N2, H7N7, H10N1, H10N7, H10N9, H11N9, and H13N9). Whether northern shoveler and Eurasian green-winged teal were also the most frequently sampled bird species in Egypt is unknown, as most larger-scaled surveillance studies in Egypt do not provide a list of the number and bird species sampled; just two [15,50] out of seven [48,49,51–53] surveillance studies provided bird species and number sampled.

In addition to northern shoveler and Eurasian green-winged teal, LPAI viruses have been isolated from northern pintail (*Anas acuta*), mallard (*Anas platyrhynchos*), rock dove (*Columbia livia*), Eurasian coot (*Fulica atra*), whiskered tern (*Chlidonias hybrida*), Egyptian Goose (*Alopochen aegyptiaca*), great egret (*Ardea alba*), and Black Kite (*Milvus migrans*) (Table 1). Based on serology of wild birds sampled in Egypt, cattle egret (*Bubulcus ibis*), hooded crow (*Corvus cornix*) and Eurasian coot may be susceptible to H5, H7 and/or H9 AIV infection [54]. In addition to LPAI viruses, HPAI H5N1 and H5N8 viruses have been isolated from apparently healthy Eurasian green-winged teals sampled in Egypt [50,51,55]. Other wild birds sampled in Egypt from which HPAI viruses have been isolated were great egret, cattle egret, and crow (H5N1) [50]. In the Northern hemisphere, AIV dynamics typically peaks during fall migration in ducks [41–43], while in more temperate regions, AIV dynamics may be less profound [56]. In Egypt, most wild birds seem to have been sampled during September to February.

## Spillover of AIV from wild to domestic birds

Based on genetic analyses of viruses isolated from birds in Egypt, some AIV subtypes were first detected in wild birds in Egypt, while others were first detected in domestic birds. HPAI H5N1 virus of clade 2.2 was first detected in Egypt in a cloacal swab from an Eurasian green-winged teal trapped in a cage by a fisherman in the Damietta region of northern Egypt in December 2005, followed by detection of genetically closely related HPAI H5N1 viruses in domestic birds (and humans) from February 2006 onwards [51]. Genetically highly similar H5 viruses have been detected on different continents, suggesting that HPAI H5N1 viruses are able to cover enormous distances with minor genetic changes [61]. Most genetic changes in the period of 2006–2015 occurred while circulating in dense bird (poultry) populations in Egypt [1]. HPAI H5N8 virus of clade 2.3.4.4 was first detected in two Eurasian coots sampled (sick or dead) in a live bird and fish market in the Damietta region in November 2016 [3], followed by HPAI H5N8 virus detection in two apparently healthy Eurasian green-winged teals sampled at an LBM in Port Said in northern Egypt early December 2016 [55]. In early 2017, HPAI virus of the H5N8 subtype was isolated from oropharyngeal swabs from ducks sampled at backyard and commercial farms [24]. LPAI H9N2 virus was first detected in tracheal/cloacal swabs/organ samples from sick birds on a broiler or broiler-breeder chicken farm in Giza/Behera/Dakahlia/Sharkia region in northern Egypt in February 2010 [2]. Besides chicken, H9N2 virus has been detected in quail (2011, 2012, 2014, 2015), turkey (2013, 2016), duck (2014, 2016) and dove (2014) (NCBI GenBank: https://www.ncbi.nlm.nih.gov/). From a global perspective, H9 is a LPAI virus subtype rare in wild ducks, but commonplace in domestic birds [17,35,62]. Remarkably, to our knowledge, no H9N2 viruses have been detected in wild birds sampled in Egypt so far. Thus, HPAI H5N1 and H5N8 viruses may have been introduced into Egypt through migratory waterfowl, while there is so far no evidence of migratory waterfowl being involved in LPAI H9N2 virus introduction into Egypt. 

Backyard poultry is a common practice in Egypt allowing interactions between wild and domesticated birds throughout the country [63]. Biosecurity and hygiene of commercial farms is generally good, but is poor in the abundant backyard farms where domestic birds roam freely in the landscape [64]. Backyard poultry have direct contact with wild birds as they leave the farm household and go outside to feed and swim. Contact rates between wild and domestic birds in Egypt have been investigated inside villages and in surrounding agricultural fields as part of a cross-sectional study, but could not be linked to HPAI H5N1 virus presence in domestic birds as number of HPAI H5N1 virus detections were too low [52]. However, mixed species and various age groups (e.g. broiler and layer farms combined, or chicken/duck/goose/turkey) have been associated with higher AIV prevalence [15,65,66]. Furthermore, the presence of waterfowl in backyards was associated with more HPAI H5N1 virus detections [15]. From backyard farms, dead birds and faeces are moved to garbage piles (until burning) in the neighborhood, a practice which has been identified as a significant risk factor for HPAI H5N1 in backyard farms [52] and presents an abundant HPAI H5N1 virus source for feral birds [12].

In addition, widespread bird hunting and trade of both live and dead wild and domestic birds allow for a considerable interface between wild and domestic birds and humans [67]. Many people–in particular fishermen – depend on wild bird capture as a source of income and protein, and to a lesser extent for recreational use. Egypt has a long history of bird hunting ranging back to the era of the pharaohs [67,68]. Despite legislation restricting bird hunting, illegal bird hunting is widespread and a total of 5.4 million birds are estimated to be killed illegally in Egypt annually [68]. Bird hunting is the most extensive in northern Egypt in the Nile Delta and its surroundings. The prime season for bird hunting is August-November, which is the season of the avian fall migration. During this season mist nets are put up along the Mediterranean shoreline to catch quails and orioles, but catch all sorts of birds heading for a rest at the sand dunes along the shoreline. Additionally, special made mansaabs (traditional tent-like structures of e.g. reeds) are put up to catch ground dwelling gallinaceous birds (e.g. corncrakes and quails). Larger birds as storks and herons are shot by rifles. Wild birds are trapped and traded – preferably alive – at local or regional LBM [12,67]. These live, apparently healthy birds (e.g. northern shoveler, Eurasian green-winged teal and Eurasian coot) have shown to be infected with HPAI viruses while sampled at a LBM [51,55]. Yet, the same LBM can be used to trade domestic birds of different species and various ages from backyards and commercial farms at several locations. Most domestic Anatidae are produced at backyard farms (64% of ducks and all geese), whereas the majority of chickens (63%) are produced at commercial farms [13]. At LBM that included waterfowl (and/or turkeys) HPAI H5N1 virus was more prevalent [15]. In summary, given the low biosecurity measures at abundant backyard farms, and the LBM where both wild and domestic birds are brought into close proximity, one could consider wild-domestic birds as one single group consisting of many populations with different susceptibilities to AIV.

## Recommendation: evidence-based measures needed to improve prevention and control

Since the introduction of HPAI H5N1 virus into Egypt early 2006, the virus became endemic in different domestic bird populations in Egypt with the highest confirmed human cases worldwide [1]. In addition, LPAI H9N2 virus was introduced into Egypt and became endemic in Egypt in 2010, and more recently in 2016 HPAI H5N8 virus was introduced into Egypt [5].The endemic situation of HPAI H5N1 and LPAI H9N2 viruses in domestic birds and diversity of LPAI viruses in Egypt are worrisome; as novel AIV could emerge that may result in more outbreaks in the future. Several factors potentially have contributed to the introduction and persistence of the AIVs in Egypt. Wild birds in Egypt can harbor a high LPAI virus diversity and may have introduced HPAI H5N1 and HPAI H5N8 viruses into Egypt, however their role in the endemicity of HPAI H5N1, LPAI H9N2 and potential other viruses – by means of genetic reassortment – is less clear. As most literature on AIV in Egypt focuses on the HA and NA of HPAI H5N1 and LPAI H9N2 viruses, little is known about AIV subtype and internal gene diversity in domestic and wild birds in Egypt. Surveillance programs, in both domestic and wild birds, that include all LPAI virus subtypes and full genome sequencing, are needed to determine the magnitude of the wild-domestic bird interface. The knowledge on AIV sub- and genotypes circulating in wild and domestic birds, in addition to HPAI H5 and LPAI H9N2 viruses, forms the basis to define the scale of the problem and to enable actions to limit and prevent AIV transmission between wild and domestic birds.

Evidence-based measures aiming to limit and prevent AIV transmission between wild and domestic birds may include interventions that minimize the contact of domestic birds with wild birds at backyard farms (such as strengthening biosecurity measures [69]) as well as around LBM (such as temporally closure [70], reduction in the illegal wild bird hunting [67], limiting markets in coastal cities [71,72], and minimizing transportation of live birds to and from LBM within and between governates [12]).

Lastly, besides wild bird-related factors, other factors such as those related to climate [29] and, in particular, those related to the poultry industry (including vaccination coverage and efficacy [73] and improper disposal of poultry carcasses [52]) shape the AIV endemicity in Egypt. Hence, evaluation of vaccination coverage and vaccine efficacy under field condition using various analysis as the cumulative annual flock immunity (CAFI) and its correlation with the virus load should be considered. In addition, it is the responsibility of both the government and the public to disseminate knowledge and regulations on the appropriate handling of both domestic and wild birds in order to decrease transmission between birds and humans.
